# Right Hemisphere Lateralization in Neural Connectivity Within Fronto-Parietal Networks in Non-human Primates During a Visual Reaching Task

**DOI:** 10.3389/fnbeh.2018.00186

**Published:** 2018-10-02

**Authors:** Jeyeon Lee, Hoseok Choi, Kyeongran Min, Seho Lee, Kyung-Ha Ahn, Hang Joon Jo, In Young Kim, Dong Pyo Jang, Kyoung-Min Lee

**Affiliations:** ^1^Department of Biomedical Engineering, Hanyang University, Seoul, South Korea; ^2^Department of Neurologic Surgery, Mayo Clinic, Rochester, MN, United States; ^3^Samsung SDS Ltd., Seoul, South Korea; ^4^Department of Neurology, Seoul National University, Seoul, South Korea; ^5^Department of Neurology, Mayo Clinic, Rochester, MN, United States

**Keywords:** fronto-parietal network, visually guided reaching, right hemisphere lateralization, non-human primates, connectivity

## Abstract

A fronto-parietal network, comprised of the posterior parietal cortex (PPC) and the dorsal premotor cortex (PMd) has been proposed to be involved in planning and guiding movement. However, the issue of how the network is expressed across the bilateral cortical area according to the effector's side remains unclear. In this study, we tested these questions using electrocorticographic (ECoG) recordings in non-human primates and using a simple visual guided reaching task that induced a left or right hand response based on relevant cues provided for the task. The findings indicate that right hemisphere lateralized network patterns in which the right PMd was strongly coordinated with bilateral PPC immediately after presentation of the movement cue occurred, while the coherence with the left PMd was not enhanced. No difference was found in the coherence pattern between the effector's side (left hand or right hand), but the strength of coherence was different, in that animals showed a higher coherence in the right hand response compared to the left. Our data support that right lateralization in long-range phase synchrony in the 10–20 Hz low beta band is involved in motor preparation stage, irrespective of the upcoming effector's side.

## Introduction

A visually guided movement requires a transformation from sensory reference frames to motor-relevant reference frames and integration between the goal related information from the prefrontal cortex (Gallivan and Culham, 2015). To achieve this, flexible and dynamic communication between task-dependent cortical regions is needed, but the underlying inherent neural mechanisms responsible for regulating these communications remain poorly understood.

Traditionally, a fronto-parietal network, comprised of the posterior parietal cortex (PPC) and the premotor cortex in the frontal lobe, is thought to be involved in the planning and guiding of movements (Picard and Strick, 2001; Fogassi and Luppino, 2005; Chouinard and Paus, 2006; Culham et al., 2006). Several recent studies reported that sub-regions of the parietal area represent the movement effector(Medendorp et al., 2005; Beurze et al., 2007, 2009; Gallivan et al., 2011a; Heed et al., 2011), the grip and transport component (Cavina-Pratesi et al., 2010) and the type of motor act (Gallivan et al., 2011b). In the case of a reaching movement, neurons on the medial bank of the PPC [including the parietal reach region (PRR), the medial intraparietal area (MIP), and the ventral area 5] and the dorsal premotor cortex (PMd) tend to respond before arm movements in monkeys and humans (Snyder et al., 1997; Wise et al., 1997; Colby, 1998; Hoshi and Tanji, 2007; Cisek and Kalaska, 2010). Pesaran et al. firstly reported that the beta-band (around 15 Hz) neural coherence between spikes and the local field potential (LFP) in the PMd and PPC in the right hemisphere, increased transiently after the search array onset and was greater when monkeys were freely making choices than when animals were following instructions (Pesaran et al., 2008). The authors suggested that the enhancement in beta coherence within the fronto-parietal circuit right after the task relevant cue presentation was related to motor planning and decision making. However, a previous report indicated that left hand reaching movement was limited to within the right hemisphere (right premotor and right parietal). The above conclusions might be based on a common assumption that the brain of non-human primates is symmetrical in function, despite the well-established hemispheric asymmetry in human brains. As a result, the issue of how the visual guided motor planning related network is expressed across the bilateral area according to effector's side continues to be unclear.

Thus, in this study, we attempted to identify the neural connectivity across the bilateral hemisphere during the visual guided reaching movement. For this purpose, we implanted two 32-channel multi electrode arrays in the epidural space of two monkeys and trained them to perform a simple visually guided reaching task to determine whether the functional connectivity was modulated by an inherent movement preparation stage and the network can be differentiated according to the hand (effector)'s side.

## Materials and methods

### Subjects and surgical procedures

**Figure 1:**
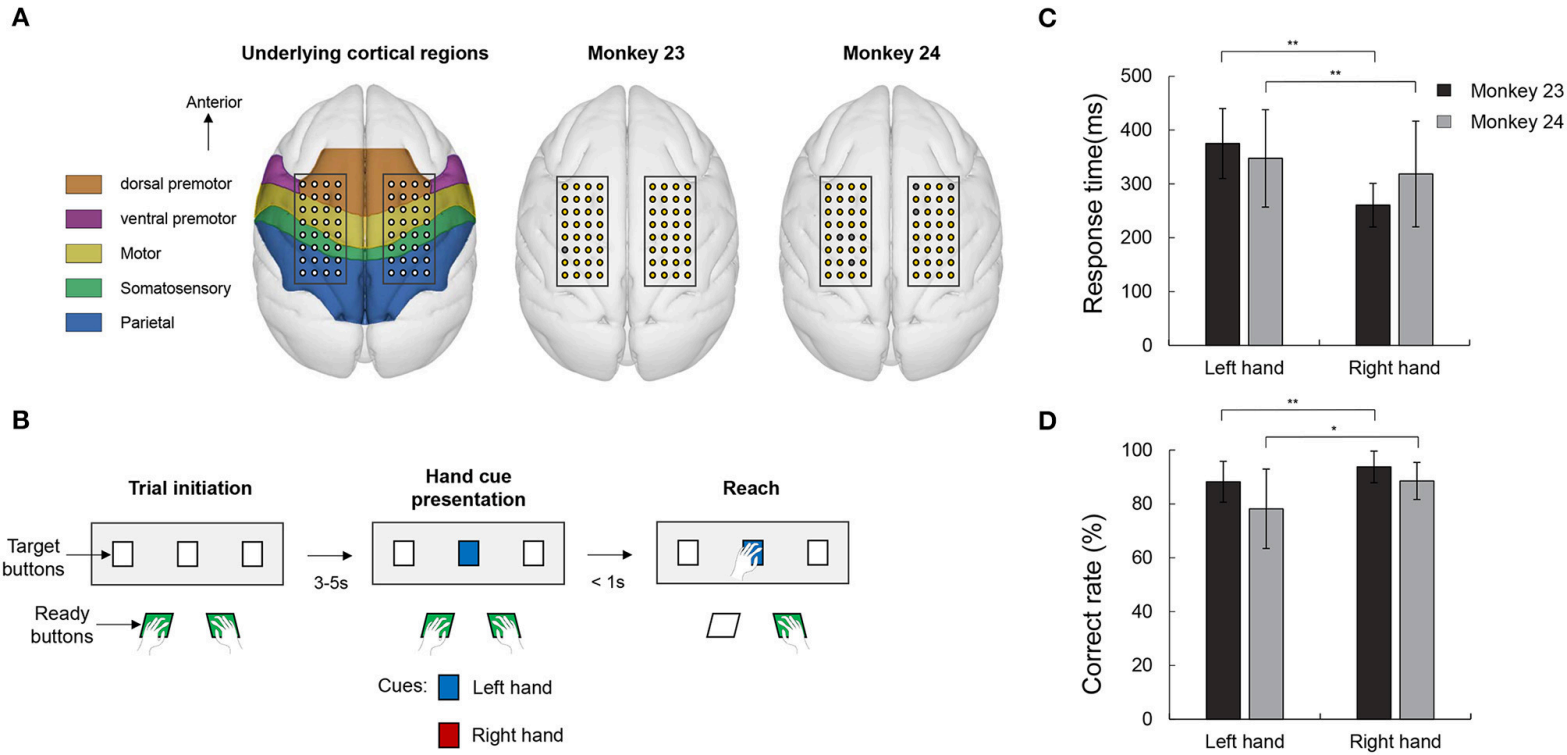
Electrode position, visually guided reaching task and behavioral results. **(A)** Electrode position and underlying cortical regions. The electrode patches were implanted in the epidural space covering PMd, M1, S1, and part of PPC. Right two panels show the location of the bad channel in both monkeys. Gray colored circles indicate the bad channels (1 channel in monkey 23 and 6 channel in monkey 24). **(B)** Visual guided reaching task. The monkeys were trained to respond with either the left or right hand according to a color cue (blue or red) presentation. **(C)** Response time in both monkeys. Both animals consistently showed an asymmetrical response in which the right hand responses were faster than the left hand (paired *t-*test, **p < 0.005). **(D)** Correct rate. Both monkeys' correct rate for the right hand trials were higher than the left hand response (paired *t-*test, **p < 0.005 and *p < 0.05).

All experimental procedures were approved by the Seoul National University Hospital Animal Care and Use Committee (IACUC No.13-0314). Two adult male rhesus monkeys (*Macaca mulatta*, Monkey 23 and Monkey 24) were included for the experiments. Two 32-channel platinum ECoG electrode arrays (Neuronexus, USA) were implanted in each hemisphere of the monkeys, covering from the PMd to part of the PPC (Figure 1A; Markov et al., 2012). The diameter of the ECoG electrodes was 300 μm and the inter-electrode distance was 3 mm. All electrodes were implanted in the epidural space. ECoG electrodes were connected to connectors (Omnetics, USA) affixed to the skull with dental cement and titanium screws (Vet implants, USA). Customized titanium head holders were also implanted to fix the monkey's head in place.

### Experimental design

The monkeys were trained to perform a simple visual guided reaching task paradigm (Figure 1B). In each experiment, the monkey was seated in a customized primate-chair, and head movement was restrained by a head holder. We installed a total of five buttons in front of the monkey for the behavioral task. Two buttons, which were placed close to the chair, were used for ready indicators and three target buttons, located around 30 cm away from the monkey, were used to indicate the target. The trial began when the animal pushed the ready buttons. After a few, randomly spaced seconds (3–5 s), while the monkey was holding the ready buttons, the target button light was then lit. The target button could flash in two colors, i.e., red or blue, each color was associated with the hand that the monkey should move. For example, if the target button flashed red, the monkey should react using its right hand and, accordingly, its left hand for the blue light. The monkeys were instructed to reach and push the target button within 1 s. If the monkey reached the target on time using the correct hand, the trial was finished and a juice reward was given. The mapping between color and hand was maintained constant throughout the training and experiments and the order of the stimulus presentation was randomized. The experiment (1 session) was conducted once a week and within a session, the monkeys performed 200~400 trials, depending on the condition of the monkey on that day. There were 11 sessions for monkey 23 and 23 sessions for monkey 24, respectively.

### Data acquisition and preprocessing

Electrical recordings started 2 weeks after recovery from the surgery. ECoG signals were digitized at a sampling rate of 1 kHz (EEG 1200, Nihon Kohden, Japan). Each button's events (press or release) were stored in the system for triggers. The movement of the monkey was also simultaneously captured at a sampling rate of 20 Hz by a wireless motion tracking system (Xsens, The Netherlands). For all experiments, each monkey wore a custom-made jacket with motion trackers affixed to the left and right shoulders, elbows, and wrists.

The whole data were analyzed offline using a customized version of Matlab (The Mathworks, Natick, MA, USA) scripts using functions from the EEGLab open-source toolbox. The data were cleaned from possible line noise artifacts with a notch filter at 60 Hz and higher harmonics (120 and 180 Hz) and a band-pass filtered from 1 to 200 Hz. Using visual inspection, channels that did not clearly contain ECoG signals (e.g., such as channels that contained flat signals or noise due to broken connections) were removed prior to the analysis. Overall, these procedures reduced the total number of channels to 63 and 58 in monkey 23 and 24, respectively (Figure 1A). We then divided the continuously recorded data into non-overlapping analysis epochs with a 2,000 ms length from 1,000 ms before the target cue light to 1,000 ms after. Bad trials containing excessive noise and artifacts were manually excluded. Moreover, we only included the correct trials in the further analyses.

### Estimation of phase synchronization

To define the connectivity between separate electrodes, we adopted the weighted phase lag index (wPLI) (Vinck et al., 2011). The wPLI is an extension of the phase lag index (PLI) which defines connectivity as an absolute value of the average sign of phase angle differences between two channels. The wPLI modified the PLI by deweighting vectors using the imaginary component of the cross-spectrum as a factor. Using this procedure, phase differences around zero or 180° (closer to the real axis) contribute only marginally to the final connectivity estimate. The wPLI can reduce the probability of detecting “false positive” connectivity where volume conducted noise sources have near zero and 180 phase lags and increases the sensitivity in detecting interactions when the interacting sources are spatially close (Vinck et al., 2011; Ewald et al., 2012; Haufe et al., 2013).

$$\text{wPLI}=\left|\frac{\sum_{i\;=\;1}^{N}Im(X_{i})}{\sum_{i\;=\;1}^{N}\left|Im(X_{i})\right|}\right|=\;\frac{\left|\frac{1}{n}\sum_{i\;=\;1}^{N}\left|Im(X_{i})\right|sgn(Im(X_{i}))\right|}{\frac{1}{n}\sum_{i\;=\;1}^{N}\left|Im(X_{i})\right|}$$

where *X*_*i*_ is the cross spectrum of two signals in trial *i* of total trial *N, sgn* is the sign of variables and *Im* is the imaginary part of complex value.

To estimate the wPLI, the data were converted to the time–frequency domain via convolution with a family of complex Morlet wavelets, defined as Gaussian-tapered complex sine waves. We used 39 linearly spaced frequencies between 2 and 40 Hz, and the number of cycles was increased from 3 to 12 in logarithmic steps (Cohen, 2015). Convolution was performed via frequency-domain multiplication, in which the Fourier-derived spectrum of the EEG data was multiplied by the spectrum of the wavelet, and the inverse Fourier transform was taken. Power and phase were defined as the squared magnitude of the complex result and the angle relative to the positive real axis respectively.

### Statistical analysis

To test whether the phase synchronization was significant under individual conditions, we applied jack-knife leave-one-out estimates of the standard error of wPLI and used the estimated standard errors to calculate significance with regard to the normal distribution of a mean of 0 and a standard deviation of 1 (Efron and Efron, 1982; Efron and Tibshirani, 1998). The *p*-values were then corrected for false discovery rate (FDR) in the time-frequency dimensions using parameters proposed by previous study (Genovese et al., 2002) with a Q = 0.2. Statistical masks were generated for corrected values of ^*^p < 0.05, a one tailed (which is appropriate for a one-sample-location test used for the wPLI that ranges from zero to one). For further analysis, we only included the significant values based on statistical mask.

## Results

### Behavioral performance of the reaching task

We firstly collected behavioral data on the monkeys. During the 34 recording sessions (11 for monkey 23 and 23 for monkey 24), the number of correct trials was 1,445/1,597 (left and right) in monkey 23 and 2,748/2,893 in monkey 24. Response time was measured as the time elapse from the cue presentation to the time when the monkey executed movement. As shown in Figure 1C, both the right hand responses (260.54 ± 40.43 and 317.85 ± 98.11 ms) were faster than the movement of the left hand (375.14 ± 64.97 and 343.55 ± 90.40 ms) (paired *t-*test, *p* < 0.005). These asymmetrical aspects can also be seen in the results for correct rate. Correct rates were calculated as an average of the performance of each session. As shown in Figure 1D, both monkeys showed a higher performance for the right hand (93.76 ± 7.60 and 88.53 ± 6.90% in monkey 23 and monkey 24, respectively) than the left hand (88.24 ± 7.10 and 78.20 ± 9.75% in monkey 23 and monkey 24, respectively) (paired *t-*test, *p* < 0.05). These results showed that, in terms of task complexity or demand, the right condition appeared to be easier for these monkeys compared with other condition.

### Phase synchronization of low-beta (10–20 Hz) frequency

**Figure 2:**
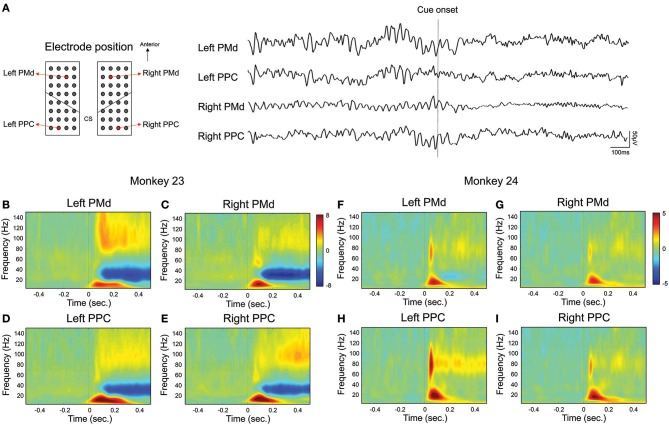
Local oscillatory activation in fronto-parietal area. Local oscillatory activity in fronto-parietal area during visually guided reaching task. For visualization, we selected representative channel pairs for the PMd and PPC across bilateral hemisphere during the right hand condition. **(A)** shows an example of raw signal trace over bilateral fronto-parietal area in single trial. Black dotted line represents the latency of cue onset. **(B–E)** represent the spectrogram of bilateral fronto-parietal channels across trials in monkey 23 and **(F–I)** represent the spectrogram in monkey 24. Black dotted line (time = 0) represents the latency of cue onset.

In order to represent local neuronal activity during visually guided reaching movement, we calculated the event-related spectral perturbation (ERSP) across trials. Figure 2A shows an example of raw signal trace of the single trial in monkey 23 on the specific channel of the bilateral PMd and PPC shown on the left. Then, Figures 2B–I represent the ERSP results which aligned to the movement cue presentation for the channels in Figure 2A. The results showed that activation of the beta and gamma frequency band increased in both animals immediately after the movement cue was presented. Notably it was observed in both areas and in both hemisphere. We then attempted to determine whether the movement cue might modulate the functional connectivity between the epidural ECoG electrodes. To this end, we extracted the Fourier spectra from 2 to 40 Hz for each condition, and computed phase coherence activation using the wPLI. The wPLI represents the strength of phase coupling between two channels, similar to the phase locking value (PLV) or coherence (where 0 indicates no phase coupling, 1 indicates maximum phase coupling), but is not spuriously increased by volume conduction of single sources to 2 sensors or common references.

**Figure 3:**
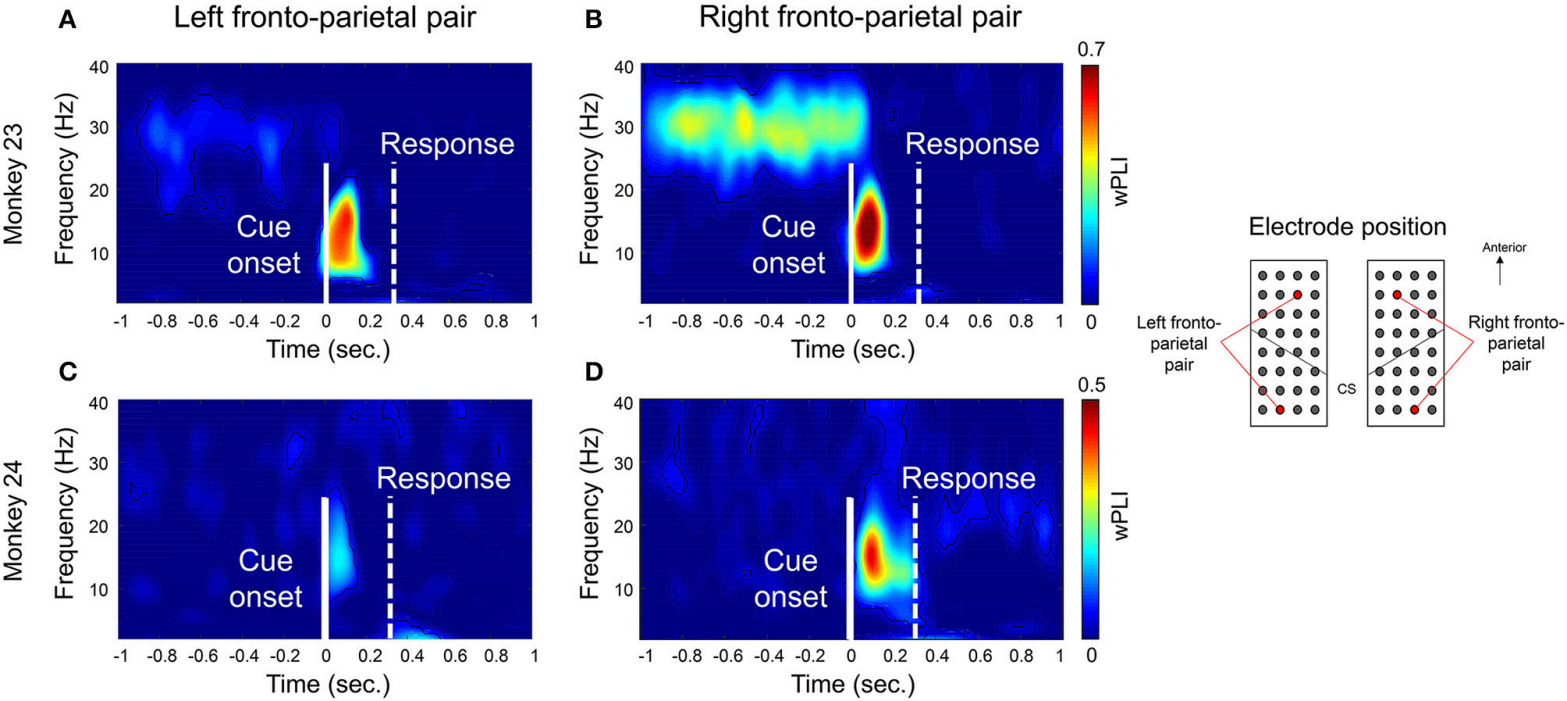
Example of 10–20 Hz phase synchronization before executing a reaching movement. Examples of time-frequency wPLI coherence. For visualization, we selected representative channel pairs between PMd and PPC across bilateral hemisphere during right hand condition. Locations of the channels were indicated in right panel. **(A,C)** show the coherence between left PMd and left PPC. **(B,D)** show the coherence between right PMd and right PPC. The solid white line indicates the task cue onset (*t* = 0) and the dotted white line indicates the average response time of the monkeys. The black contour line inside the images indicates the statistical significance (Adjusted *p* < 0.05, FDR corrected).

Figure 3 shows an example of the coherence between the bilateral PMd and bilateral PPC for both monkeys. For visualization, we selected representative channel pairs over each region (indicated in right panel in Figure 3). Consistent with previous findings (Pesaran et al., 2008), both animals showed a strong coherence at the low beta frequency band (around 10–20 Hz) between right PMd and right PPC (Figures 3B,D). The coherence emerged immediately after the cue presentation (time = 0, indicated by a solid white line) as a prominent peak near 100 ms and disappeared before the actual movement response. In contrast, the coherence in low-beta band between left PMd and left PPC was weaker than the coherence of the right pair in monkey 23 or barely observed in monkey 24 (Figures 3A,C, respectively). In monkey 23, a strong coherence of higher frequency (around 25–35 Hz) was also observed prior to the onset of the task-relevant cue and offset immediately after cue presentation, however these data were not included in further analysis due to inconsistency across animals. To test whether the coherence is related to the decisional stage or motor process, we tried to calculate the wPLI coherence by aligning the same data on the latency of movement onset. As a result, we observed that the peak value decreased compared to when the data were time-locked to the cue onset (Supplementary Figure 1). Therefore, we speculated that beta-coherence might be closer to the decisional or perceptual process of task-related cue.

**Figure 4:**
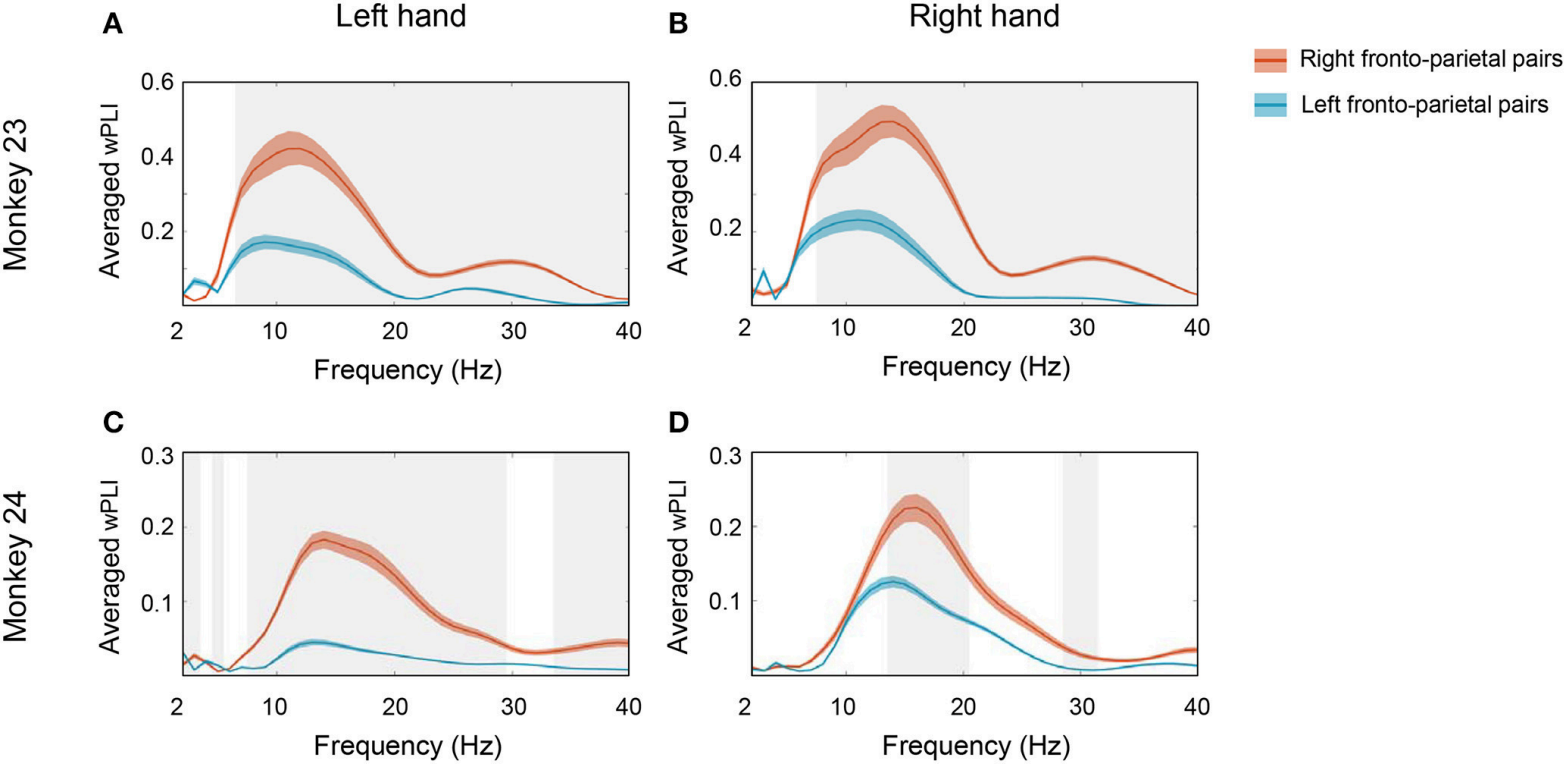
Spectral profile of the averaged wPLI. To specify where the coherence emerged in the frequency domain, we calculated the average montage of wPLI across channel pairs over bilateral PMd and PPC. The average was taken along the time window from 0 to 0.2 s. The solid red line represents the averaged wPLI values of right fronto-parietal pairs and the solid blue line represents the averaged wPLI of left fronto-parietal pairs. **(A,B)** show the spectral profile of averaged wPLI in monkey 23 in left hand and right hand conditions, respectively. **(C,D)** show the spectral profile of averaged wPLI in monkey 24 in left hand and right hand conditions, respectively. The gray shaded area represents the statistical significance between both pairs (*p* < 0.0005, *t-*test) and the colored shaded area represents SEM.

**Figure 5:**
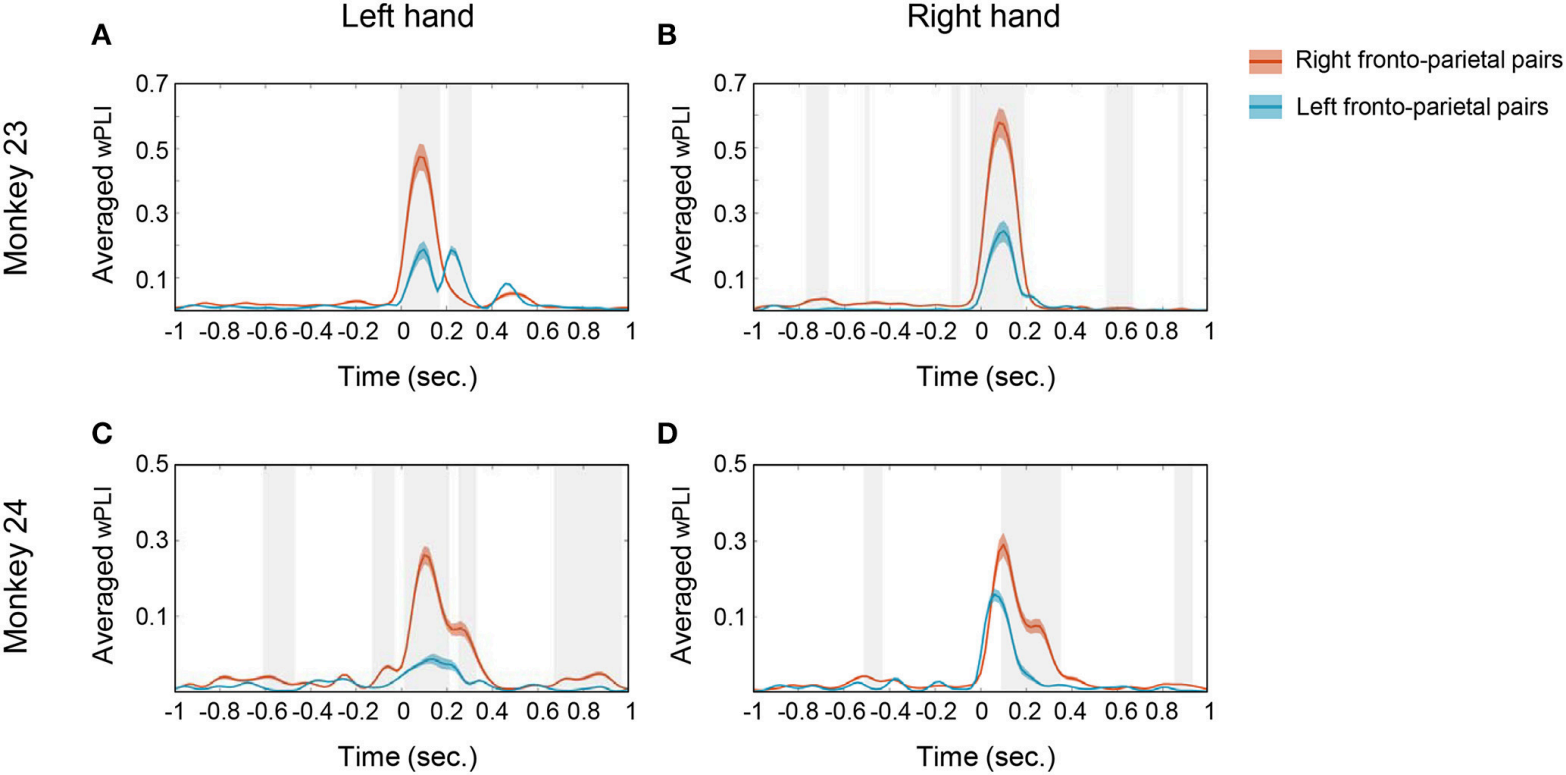
Temporal profile of the averaged wPLI. To specify the temporal profile of wPLI, the average was taken along the frequency window from 10 to 20 Hz. The solid red line represents the averaged wPLI values of right fronto-parietal pairs and the solid blue line represents the averaged wPLI of left fronto-parietal pairs. **(A,B)** show the temporal profile of averaged wPLI in monkey 23 in left hand and right hand conditions, respectively. **(C,D)** show the temporal profile of averaged wPLI in monkey 24 in left hand and right hand conditions, respectively. The gray shaded area represents the statistical significance between both pairs (*p* < 0.0005, *t-*test) and the colored shaded area represents SEM.

To summarize the overall coherence patterns across bilateral fronto-parietal network and to specify where the coherence emerged in the spectro-temporal domain, we then calculated the average montage of wPLI values along frequency and time axes between every possible pairs over each region (Figures 4, 5). Figure 4 represents the averaged wPLI in the spectral domain which was calculated from 0 to 0.2 s along time axes. Consistent with Figure 3, we confirmed that the coherence of the low beta with a prominent peak near 15 Hz was strongly enhanced in both monkeys. Moreover, we also found that the coherence of the right fronto-parietal pair was stronger than that of left fronto-parietal pair. As illustrated in Figure 4, right PMd was strongly coordinated with right PPC (red line) while the coherence between left PMd and left PPC (blue line) was relatively low in both monkeys. Interestingly, such asymmetry remained regardless of hand movement side. Whether the monkeys moved right hand or left hand, the coherence within right hemisphere was stronger than left hemisphere.

The temporal profile of 10–20 Hz coherence, illustrated in Figure 5, suggests that the temporal evolution of coherence emerged shortly after cue presentation (time = 0). Note that both monkey's mean movement execution time (indicated by the dotted white line in Figure 3) was ~300 ms; thus the coherences mostly disappeared before the actual onset of movement. Both monkey's mean response took 299.99 and 313.03 ms in monkey 23 and monkey 24, respectively (Figure 1C). This result suggests that the phase coherence in this low beta band may signify a visual perception and decision related network that is functionally activated immediately posterior to the onset of task-relevant stimuli in both animals.

### Topography of low beta phase synchronization enhancement

**Figure 6:**
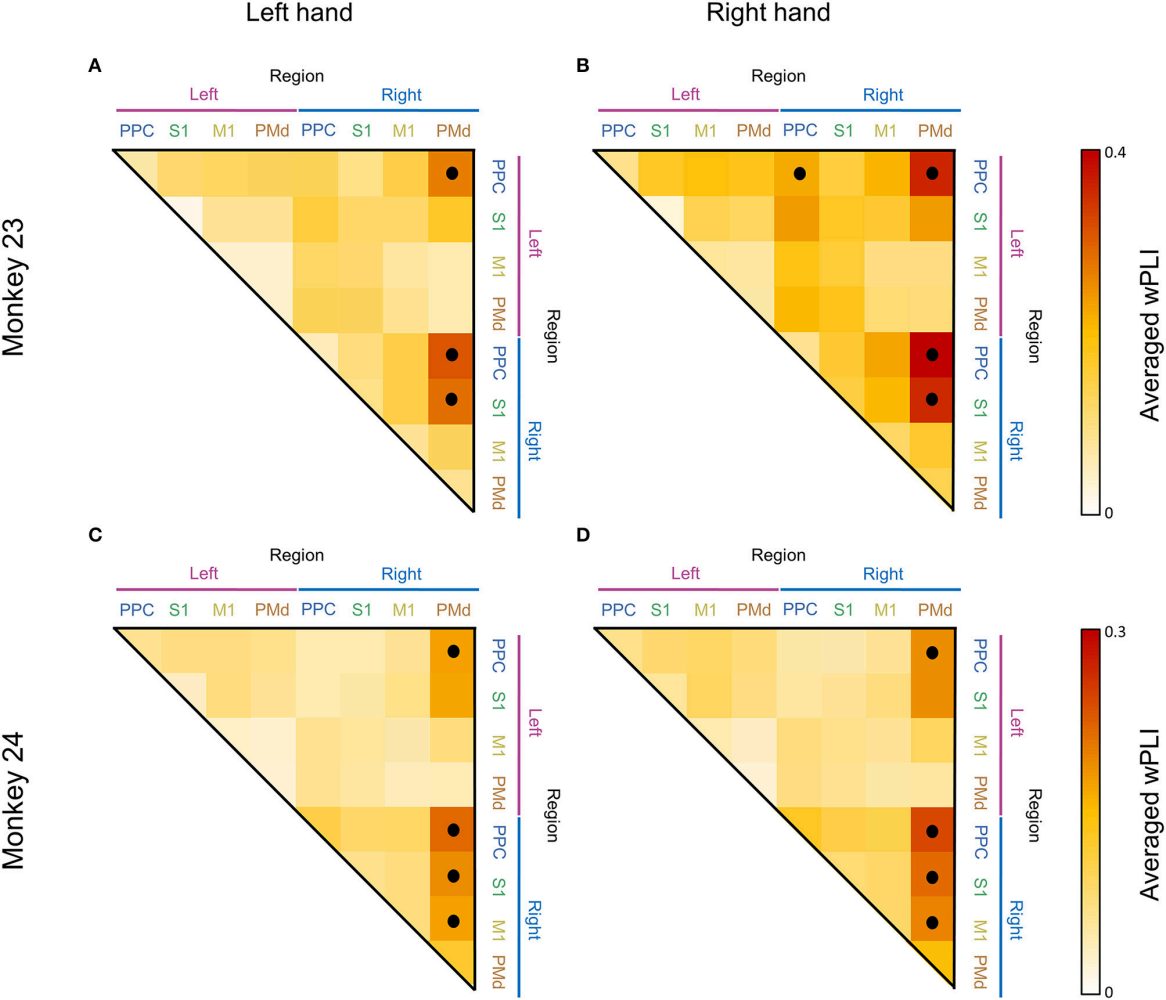
Adjacency matrix showing the average of 10–20 Hz coherence between regions. Each adjacency matrix was calculated by averaging the coherence values across corresponding electrode pairs within the specific brain regions where the averaging window was defined as 0–0.2 s and 10–20 Hz. Panels **(A,C)** show the coherence pattern before the movement of the left hand and panels **(B,D)** show the coherence before the right hand movement. The black dots represent statistical significance (*p* < 0.001, Wilcoxon signed rank test).

To illustrate a topographical overview of the low beta coherent network, we calculated the adjacency matrices during the post-cue period for all conditions (Figure 6). Each matrix was calculated by averaging the wPLI coherence values across corresponding electrode pair within the specific brain regions where the averaging window was defined as 0–0.2 s and 10–20 Hz. Only the statistically significant values (inside black contour line in Figure 3, Adjusted *p* < 0.05) were included for average calculation. We employed the Wilcoxon signed-rank test to determine whether each of the values were significantly greater than their median value to exclude the case where the whole matrix value increases due to the volume conduction effect. As result, we found a right lateralized fronto-parietal network pattern in both monkeys. Consistent across animals, the right PMd was most strongly connected with bilateral PPC, including S1 and M1 (Wilcoxon signed-rank, *p* < 0.001), while the left PMd was weakly or barely coordinated with the bilateral parietal area in both animals for all conditions (Figure 6). Additionally, to quantify the similarity of the low beta network across animals, we calculated the Pearson correlation coefficient for the adjacency coherence matrices of both monkeys. We found a strong significant correlation between the two matrices for every condition (across cells in the matrices) (*r* = 0.7539, *p* < 0.0001 and *r* = 0.6678, *p* < 0.0001 for left hand and right hand, respectively).

**Figure 7:**
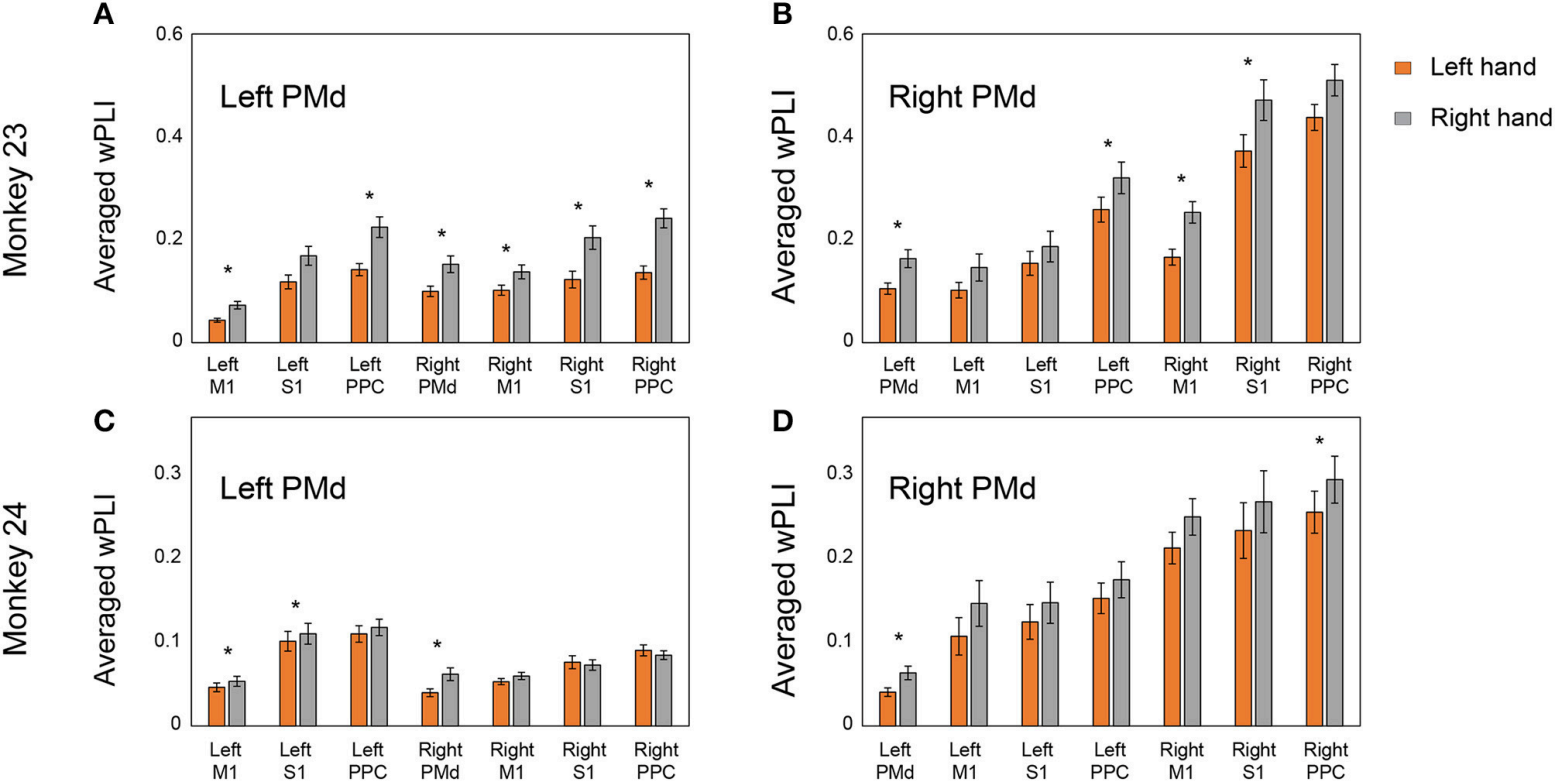
Distribution of the averaged coherence from bilateral PMd in right and left hand conditions. Panels **(A,C)** show the averaged wPLI pattern between the left PMd and other areas. Panels **(B,D)** show the averaged wPLI pattern between the right PMd and other areas. Asterisks indicate statistical significance (*p* < 0.05, Mann–Whitney *U* test). Right PMd was dominantly coordinated with both PPC areas and there was significant difference between the conditions in which the coherence of right hand condition was higher than the left hand.

We further summarized the averaged wPLI between areas using bar plots. Figure 7 shows the distribution of the averaged coherence of electrodes from each PMd to other areas. Consistent with the adjacency matrix (Figure 6), Figure 7 demonstrates the right PMd was strongly coordinated with the bilateral PPC area across both animals, while the coherence between the left PMd and bilateral PPC was not enhanced. We also compared the averaged wPLI value between the effector's side (left hand or right hand). There was a statistically significant difference for the overall coherence being higher in the right hand condition than left hand movement in both monkeys.

## Discussion

This study demonstrates the existence of a highly structured functional coherent network which was modulated by a task-relevant cue during a simple visual-guided reaching movement. This large-scale network was temporally well locked to the cue presentation, was specific to a 10–20 Hz frequency band, which corresponds to the classical low-beta frequency range, and showed a consistent topography in which the right lateralized fronto-parietal network encompasses the right PMd and bilateral PPC in both animals.

One of the main findings in our study is the identification of a right-lateralized long-range network in which the low beta-band is synchronized after an instruction cue. We provide the evidence to show that coordination between the right PMd and the bilateral PPC is prominent for the movement preparation stage, regardless of the upcoming effector's side, while coherence between the left pre-motor and parietal cortex is only marginally implicated with the hand movement. There are several reports of right lateralized brain function in humans (e.g., visuospatial function; Heilman and Van Den Abell, 1980; Shulman et al., 2010; De Schotten et al., 2011), however a right dominant pattern has not been reported in existing non-human primate electrophysiology research, and it was commonly believed that there is no inter-hemispheric difference in the brain of non-human primates. There is a limitation of this study that any claims on lateralization are difficult to make with just two subjects. This study, however, shows the possibility of the existence of right-lateralized brain function in the hand movement preparation stage in non-human primate. One possible reasons for the discrepancy might be due to the usage of phase-based connectivity in the analysis. Actually, neural activity and phase-based connectivity can be dissociated from one another. In other words, the phase synchronization between channels could not be simply explained by changes in local oscillatory activity. Previous studies have shown that connectivity strength increased, even when the actual band power was reduced (Hipp et al., 2011). Hipp et al. showed the enhanced beta-synchrony during stimulus processing was contrasted by a profound and widespread suppression of local beta-band activity. Moreover, we also showed that the local beta-band activity increased bilaterally and in monkey 24, and the power of left hemisphere was generally higher than right hemisphere on the contrary with right lateralized beta coherence (Figure 2 and Supplementary Figure 2). Therefore, there is a possibility showing asymmetrical brain connectivity regardless of brain activity.

The beta-band synchronization may serve as a general mechanism for mediating large-scale interactions across a network of frontal and parietal areas. Several recent studies demonstrated that beta synchrony was related to a large-scale network and is implicated in playing a role in perception (Lumer et al., 1998; Leopold and Logothetis, 1999; Sterzer et al., 2009) and the selective control of attention (Barceló et al., 2000; Kastner and Ungerleider, 2000; Corbetta and Shulman, 2002; Serences and Yantis, 2006). Furthermore, it has been reported that beta-band activity across frontal and parietal regions is implicated in visual attention, decision making and sensorimotor integration (Kopell et al., 2000; Gross et al., 2004; Buschman and Miller, 2007). Especially, the beta-band coherence between the right PMd and the bilateral PPC found in this study are in agreement with previously reported findings (Pesaran et al., 2008) which demonstrated that the beta coherence in the frontal-parietal circuit, especially PMd and PPC in the right hemisphere, was enhanced during the motor planning and the decision process. Consistent with previous finding, we showed that the coherence between the fronto-parietal area was specific to the beta band and emerged after cue presentation and disappeared within 200~300 ms (Figure 3). During the first 200 ms after the instruction cue, the brain signals may reflect not only the movement planning but also the other cognitive functions e.g., visual perception or attentional processing. The possibility could not be excluded with simple movement tasks in this experiments that the founded coherencies may reflect any other cognitive process as well as the motor planning and the decision process. A control trial study (e.g., free choice) would be required for the clarification of the functional roles of coherence in those brain areas could not be clear in movement preparation stage.

The difference in coherence strength between conditions may be correlated with behavior. In our behavioral results, both monkeys consistently showed a shorter response and a higher correct rate in right hand conditions than for the left hand. Several previous studies have reported that cortico-cortical connectivity is tightly correlated with the cognitive function (working memory and visual attention) or behavior performance (Hampson et al., 2006a,b; Prado et al., 2011). Given this point of view, the behavioral asymmetries that both monkeys showed might reflect a difference in network strength as shown in Figure 7. However, because the task employed in this study was too simple to permit this question to be tested, additional experiments would be needed for more in-depth evaluations.

## Author contributions

JL, HC, and K-HA performed the measurements. D-PJ and KML were involved in planning and supervised the work. JL processed the experimental data, performed the analysis, drafted the manuscript, and designed the figures. KM, SL, HJ, and IK aided in interpreting the results and worked on the manuscript. All authors discussed the results and commented on the manuscript.

### Conflict of interest statement

The authors declare that the research was conducted in the absence of any commercial or financial relationships that could be construed as a potential conflict of interest.
