# Supplementary material for: Right Hemisphere Lateralization in Neural Connectivity Within Fronto-Parietal Networks in Non-human Primates During a Visual Reaching Task
Source: Front Behav Neurosci. 2018 Oct 2;12:186. doi: 10.3389/fnbeh.2018.00186 (PMC6176198; doi:10.3389/fnbeh.2018.00186)
Supplement: Supplementary file 1 [file Image_1.pdf]

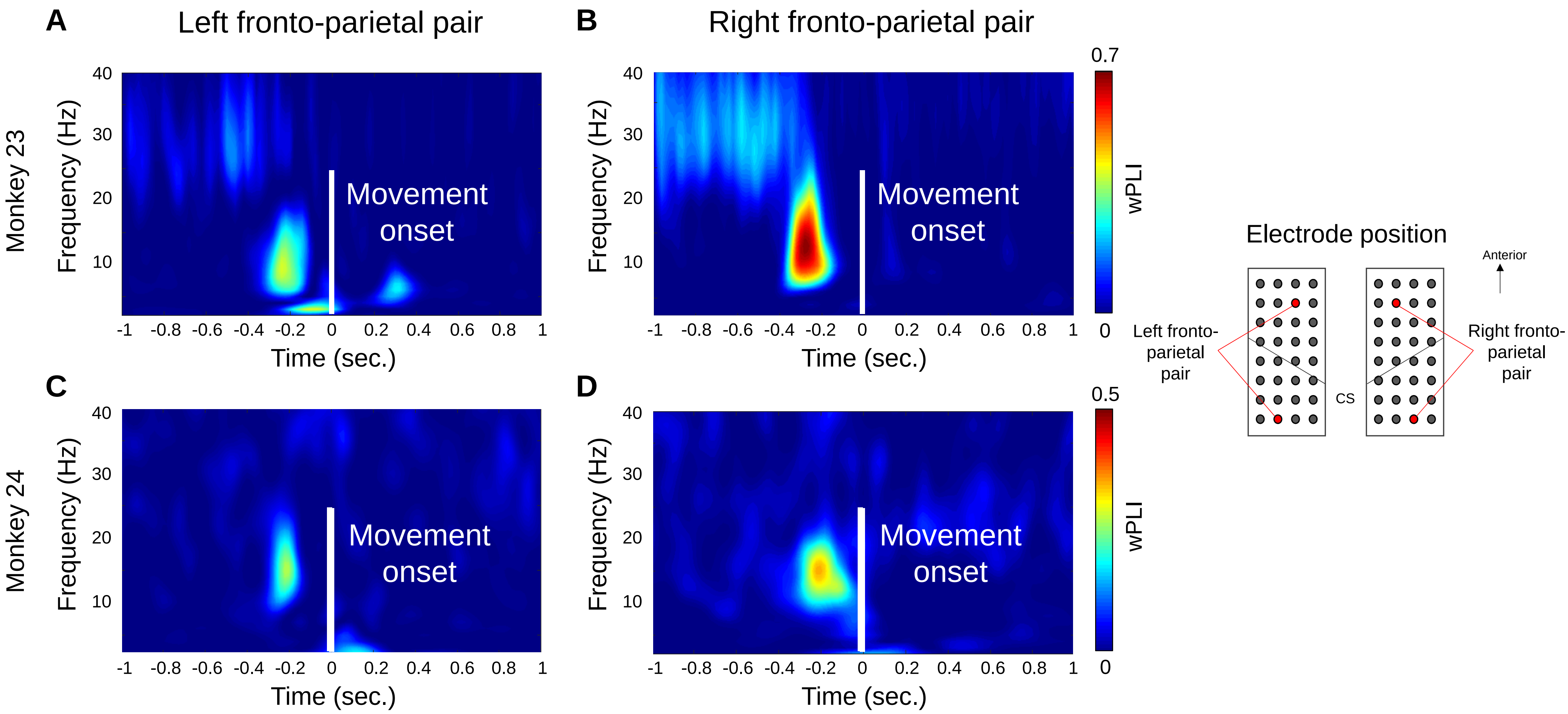

Supplementary Figure 1. Example of 10–20Hz phase synchronization aligned by movement onset. In order to test the c oherence is related to the decisional stage or motor process, we tried to calculate the wPLI coherence by aligning the sam e data on the latency of movement onset. For visualization, we selected representative channel pairs between PMd and P PC across bilateral hemisphere during right hand condition. Locations of the channels were indicated in right panel. **(A, C)** show the coherence between left PMd and left PPC. **(B, D)** show the coherence between right PMd and right PPC. Th e solid white line indicates the movement onset (t = 0). The black contour line inside the images indicates the statistical s ignificance (Adjusted $p < 0.05$, FDR corrected).
