# Supplementary material for: Right Hemisphere Lateralization in Neural Connectivity Within Fronto-Parietal Networks in Non-human Primates During a Visual Reaching Task
Source: Front Behav Neurosci. 2018 Oct 2;12:186. doi: 10.3389/fnbeh.2018.00186 (PMC6176198; doi:10.3389/fnbeh.2018.00186)
Supplement: Supplementary file 2 [file Image_2.pdf]

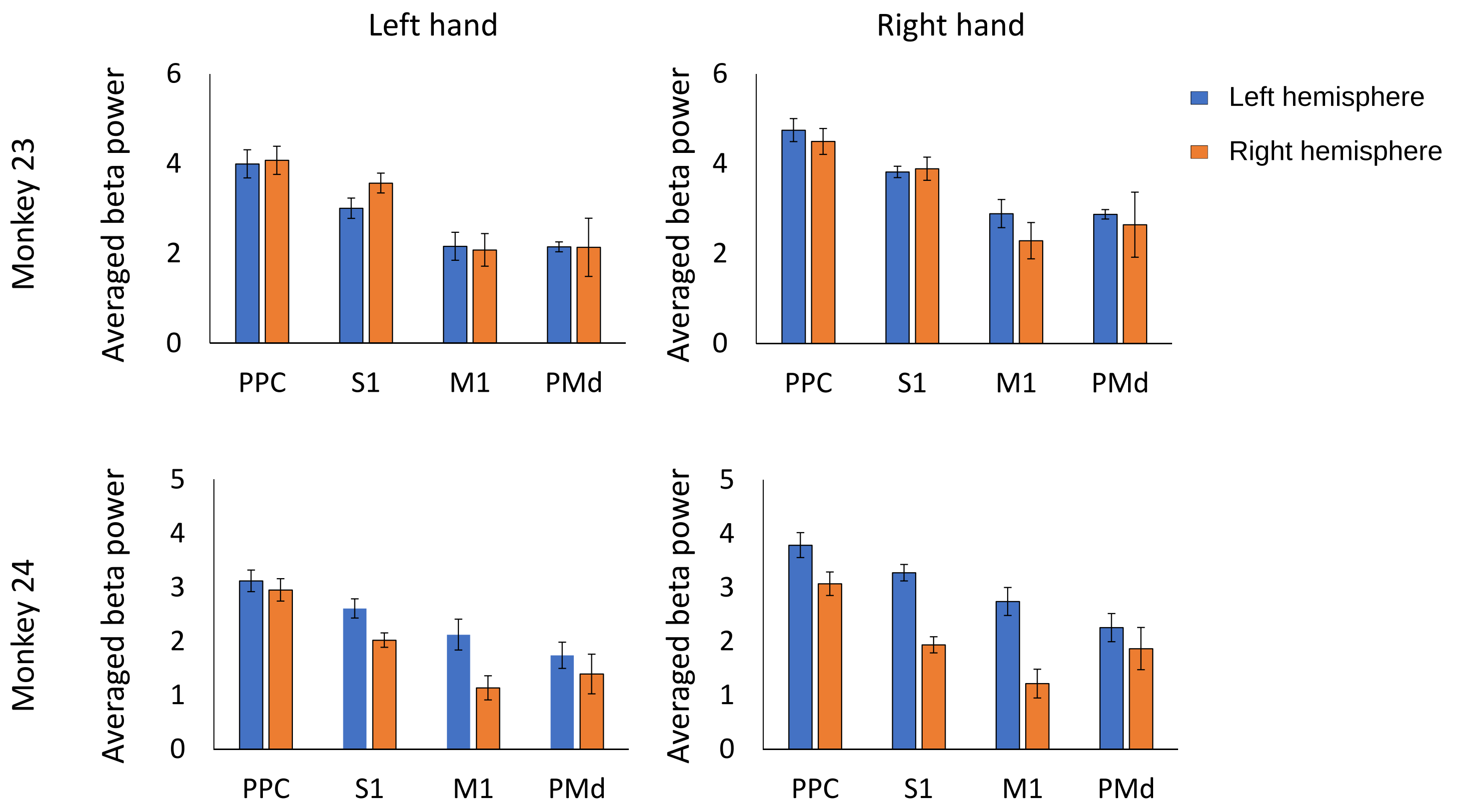

Supplementary Figure 2. Distribution of the averaged low beta power across area. For the comparison with the right lateralized pattern of wPLI coherence, the low beta band activity over channels within each cortical region were averaged where the averaging window was defined as 0–0.2 s and 10–20Hz. Error bars represent SEM.
